# Effects of Tocilizumab on Inflammation and Iron Metabolism in Critically Ill Patients with COVID-19

**DOI:** 10.3390/pharmaceutics15020646

**Published:** 2023-02-14

**Authors:** Robert Szabo, Cristina Petrișor, Constantin Bodolea, Vlad Dobre, Sebastian Tranca, Simona Clichici, Iulia Szabo, Razvan Marian Melinte, Teodora Mocan

**Affiliations:** 1 Physiology Department, “Iuliu Hatieganu” University of Medicine and Pharmacy, 400000 Cluj-Napoca, Romania; 2 2nd Anesthesia Department, “Iuliu Hatieganu” University of Medicine and Pharmacy, 400000 Cluj-Napoca, Romania; 3 Department of Anesthesia and Intensive Care, Clinical County Emergency Hospital, 400000 Cluj-Napoca, Romania; 4 Municipal Clinical Hospital, 400139 Cluj-Napoca, Romania; 5 Department of Rheumatology, “Iuliu Hațieganu” University of Medicine and Pharmacy, 400000 Cluj-Napoca, Romania; 6 Department of Orthopedics, Regina Maria Health Network, 540098 Targu Mures, Romania; 7 Department of Orthopedics, Humanitas Medlife Hospital, 400664 Cluj-Napoca, Romania; 8 Nanomedicine Department, Regional Institute of Gastroenterology and Hepatology, 400000 Cluj-Napoca, Romania

**Keywords:** COVID-19, tocilizumab, anemia, interleukin 6, hepcidin, ferritin, nanoparticles, cytokine storm, hyperferritinemia

## Abstract

COVID-19 produces cytokine-mediated persistent inflammation and is associated with elevated iron stores and low circulating iron. It is believed that central to the pathophysiological mechanism is interleukin 6 and hepcidin. A state of iron overload, termed hyperferritinemia, and inflammatory anemia take place. Both conditions are linked to a worse result in critically ill patients. Blocking the interleukin 6—hepcidin pathway with Tocilizumab could present favorable outcomes. The aim of this study was to evaluate if Tocilizumab influences survival, the occurrence of sepsis, anemia and transfusions in critically ill patients suffering from COVID-19. This prospective observational study focused on levels of interleukin 6, hepcidin and blood iron parameters in patients treated with Tocilizumab. Data were compared before and after therapy as well as between treated and control groups. Results indicate that there is no difference in terms of survival nor in the rate of anemia or sepsis occurrence. Hepcidin was elevated and anemia ensued after treatment, which could indicate alternative pathways. In conclusion, when the classic interleukin 6—hepcidin pathway is blocked, inflammation seems to use alternative routes. Further understanding of these pathways is required and new pharmacological therapies need to be developed to treat persistent inflammation.

## 1. Introduction

Iron homeostasis is influenced by factors such as iron stores and circulating plasma iron concentration. While these are physiological events, in illness inflammation and hypoxia can have an unwanted result on iron metabolism [1]. SARS-CoV2 infection produces an exaggerated and uncurbed immune response and creates a cytokine storm (CS) which then maintains inflammation [2].

Inflammation is known to produce hyperferritinemia and to reduce serum iron, resulting in anemia, both of which have profound negative effects [3,4]. Iron homeostasis is affected by hepcidin, a polypeptide synthetized in the liver, and acts on ferroportin by internalizing and degrading it [5]. Hepcidin usually has a short effect, lasting up to two days, but the constant secretion of cytokines causes a lengthy state of iron dysmetabolism [6]. In cases where inflammation is uncurbed, exaggerated iron accumulation takes place, which servs as substrate for the generation of reactive species of oxygen (ROS) and finally cell death, termed ferroptosis [7].

Hyperferritinemia was associated with increased morbidity and mortality in critically ill patients suffering of COVID-19 and was considered a negative predictor of outcome [8]. The culprit for high levels of ferritin is thought to be interleukin (IL) 6, which induces the transcription of the HAMP gene [6,9]. Tocilizumab, a monoclonal inhibitor of the IL-6 receptor, has been used in the treatment of severe cases of COVID-19 [10]. The effects on survival were analyzed in some studies and presented promising outcomes. To our knowledge, the results of direct inhibition of the IL-6 pathways have not yet been studies in terms of an effect on inflammatory anemia and the need for blood products. The aim of this study is to evaluate the effects of tocilizumab on anemia and transfusions, as well as on survival and sepsis.

## 2. Materials and Methods

### 2.1. Study Design and Setting

A cross-sectional, case–control, prospective observational study was conducted. Ethics approval from the University ethics board as well as the Hospital’s ethics committee were obtained and registered under the following references: 140/06.05.2019 and 8961/05.04.2019. Eligible cases were severe and critical patients suffering of COVID-19, who required admission to critical care unit (ICU) of the Emergency Clinical County Hospital Cluj Napoca. The inclusion period was between February and December 2020. Definition and criteria for disease severity are detailed in Appendix A [11].

### 2.2. Participants

After gaining patient consent, directly from the patient or next of kin when unconscious or sedated, all adults (18 or above) who required ICU admission for at least 48 h, were entered in the study. A detailed history and assessment were performed on all included patients to identify exclusion criteria.

Patients were excluded from the study if: refusal to participate was expressed, hemoglobin levels were <13 g/dL for males and <12 g/dL for females, history of blood transfusions during the past week, history of iron supplementation or iron lowering therapy, history of renal impairment defined by a glomerular filtration rate below 30 mL/min, history of chronic dialysis or acute renal replacement therapy before baseline assessment, history of disease producing altered metabolism of iron, expecting or nursing mothers, history of inflammatory illnesses, history of cancer and chemotherapy, viral infections other than COVID-19.

### 2.3. Variables and Data Source

All patients underwent standard bundle of care. Blood tests were obtained at three- and four-day intervals, respectively, starting on admission and continued up to twenty-eight days. All patients were treated with corticosteroids (dexamethasone 6 mg/day) for ten days, remdesivir if within seven days from initial symptoms and no contraindications were present (liver or renal impairment), favipiravir in cases where remdesivir was contraindicated or not available. Based on blood tests, tocilizumab 8 mg/kg (to a maximum of 800 mg) was administered once if titers of IL-6 were >100 pg/mL and infections were ruled out. Apart from bacterial, viral infections such as hepatitis, HIV and cytomegalovirus were also ruled out prior to IL-6 blockade. Major immunosuppressing social conditions such as chronic alcohol use or drug abuse were also ruled out before treatment. All patients received prophylactic or curative doses of enoxaparin, depending on clinical indication. Patients were followed up to 28 days from admission or less, if death or discharge ensued before the full period elapsed. The aim of the study was to follow patients who received treatment with tocilizumab, for primary and secondary endpoints. The primary endpoints were the occurrence of events such as death, sepsis, anemia, and transfusions of red blood cells during admission. Sepsis was considered when the sequential organ failure assessment (SOFA) score was two or more points and a suspicion for infection was present concomitantly, upheld by either clinical suspicion, a pathogen identified, radiological findings suggesting an infection or polymorphonuclears on sputum gram staining [12]. Anemia was present when hemoglobin <12 g/dL in females and <13 g/dL in males [13]. Secondary outcomes were changes in hemoglobin, serum iron, transferrin saturation, transferrin, ferritin, hepcidin, IL-6, leucocytes, C reactive protein (CRP) and procalcitonin levels after treatment with tocilizumab.

To assess if the effects were produced by tocilizumab, controls were matched by age, sex, and comorbidity. The control group was comprised of patients from a cohort enrolled for a concomitant study during the same period [14]. Patients in the control group followed the same inclusion and exclusion criteria. For the secondary outcomes, patients were their own control. In the treatment group the values before treatment and 7 days after treatment were analyzed. In the control group, values from admission and day seven were chosen. Routine parameters were determined in the hospital’s accredited laboratory while hepcidin and IL-6 were outsourced and determined from serra. Human interleukin 6 and hepcidin ELISA kits were purchased from Quantikine. The APACHE II (Acute Physiology and Chronic Health Evaluation II) and Charlson Comorbidity Index scores were used to quantify comorbidity.

### 2.4. Statistical Methods

Values of blood parameters on admission were compared between controls and cases while for treated patients, values before receiving tocilizumab were compared with values from a week after treatment. Data were first analyzed for normality of distribution using Kolmogorov–Smirnov and Shapiro–Wilk tests. *t*-tests for parametric data and Mann-U for non-parametric data were selected. Values were expressed as mean and standard deviation or median with range. For comparing time to an event between cases and controls, Kaplan Meier survival curves were analyzed, and results expressed as hazard ratio and median survival time. Categorical data were analyzed using Fisher’s exact test. For statistical significance, a cutoff value for *p* < 0.05 was selected.

## 3. Results

**Figure 1:**
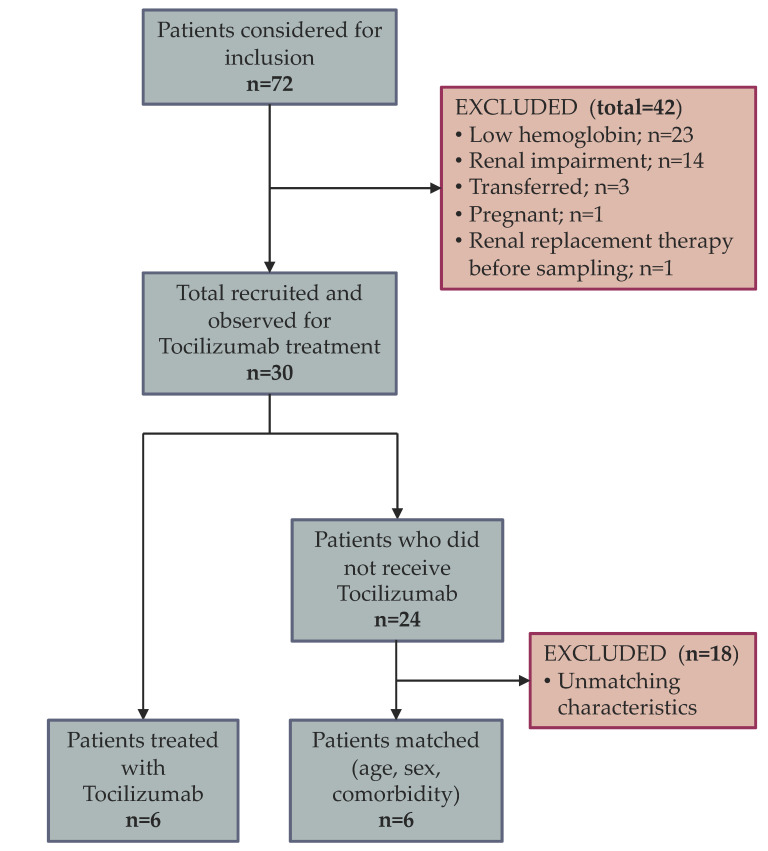
Flow of patients from analysis to inclusion and matching.

In the study timeframe, 72 patients were admitted and criteria for inclusion as well as exclusion were evaluated. A total of 42 patients were excluded. The exclusion criteria and the flow of patients are displayed in Figure 1. After recruitment, 30 patients were followed for indications and treatment with tocilizumab. Treatment was administered to six patients. The remainder of 24 patients were analyzed for age, sex and comorbidity and a selection of six matched controls was carried out.

Baseline characteristics and laboratory parameters were obtained and analyzed on admission. Results are seen in Table 1. No differences were observed between patients treated with tocilizumab and controls, on admission to ICU.

**Figure 2:**
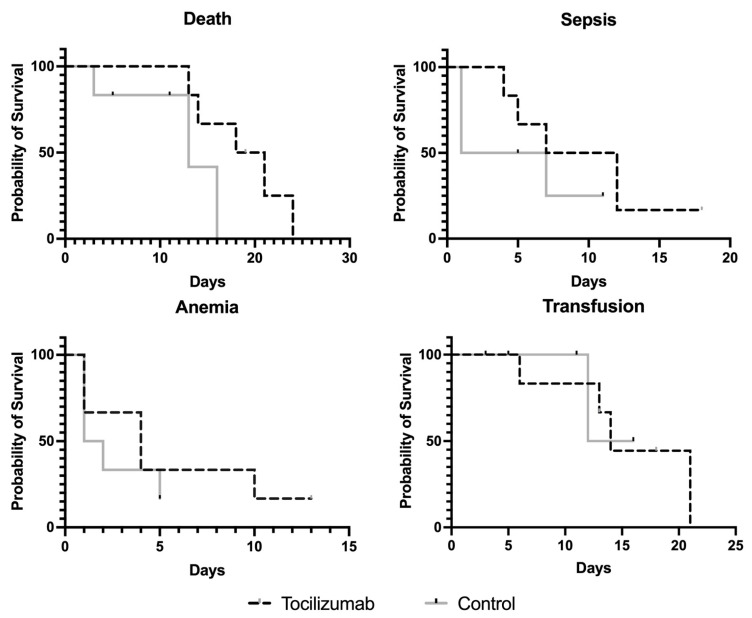
Kaplan Meier analysis of true survival, sepsis, anemia, and transfusion in patients treated with tocilizumab and their matched controls.

Time to an event was analyzed in controls and the treated group, with results shown in Figure 2. Controls presented a median survival period of 13 days compared to treated patients where median survival was 19 days and a HR of 6.532 (CI 0.7991–53.39), however the statistical significance was not reached (*p* = 0.08). When considering sepsis as an event, controls and treated patients presented similar median survival time of 4 and 9 days, respectively, with a HR of 2.21 (CI 0.426–11.36; *p =* 0.346). For occurrence of anemia, median survival was 1 day in the control group and 4 days in the treated group with a HR of 1.733 (CI 0.376–7.984). The rate of event occurrence was not statistically significant (*p =* 0.481). Finally, tocilizumab did not influence median survival to transfusion with 14 days in both control and treatment groups (HR of 0.954 CI 0.1–9.131; *p =* 0.967).

**Figure 3:**
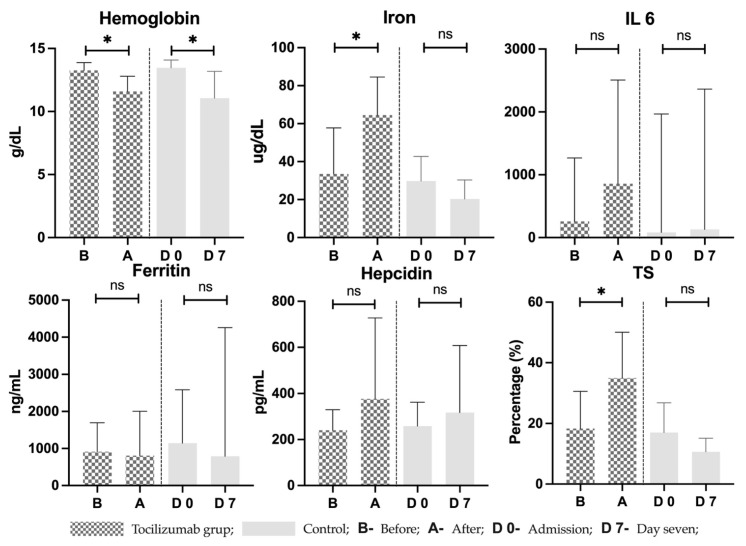
Laboratory parameters in tocilizumab and control groups. B—right before receiving treatment with tocilizumab; A—seven days after treatment with tocilizumab; D 0—day of admission in control group; D 7—day seven after admission in control group; TS = transferrin saturation; IL-6 = interleukin 6; * = statistically significant; ns = statistically non-significant.

Hematological laboratory parameters were analyzed on admission and day seven in controls as well as before receiving tocilizumab and seven days after (Figure 3). Hemoglobin decreased significantly in both groups, with a mean of differences of −1.667 ± 1.371 g/dL (*p =* 0.031) in treated and −2.400 ± 0.882 g/dL (*p =* 0.03) in controls. Iron studies showed that while an increase in circulating iron was observed in the treatment group, in the control group this registered an an opposite trend. Serum iron increased by a mean difference of 31 ± 12.85 µg/dL (*p =* 0.037) in the tocilizumab group and decreased by a mean difference of −9.5 ± 8.61 µg/dL (*p =* 0.31) in the control group, with the latter not reaching significance. TSAT also increased in the treated by a mean of 16.67 ± 15.25% (*p =* 0.044) but followed a decreasing trend in controls by a mean difference of −6.333 ± 6.122% (*p* = 0.33). There were no significant differences in the groups for neither ferritin (*p =* 0.97 in treated and *p =* 0.66 in controls) nor transferrin (*p =* 0.62 in treated and *p =* 0.47 in controls). IL-6 followed an increasing trend in both the treatment and control groups, with a mean difference of 674.6 ± 838.9 pg/mL in the treated and 341.3 ± 554.5 pg/mL in controls; however, this did not reach statistical significance (*p =* 0.106 in treated and *p =* 0.552 in controls). Hepcidin was also unchanged with a marginal mean difference of 136.0 ± 377.2 ng/mL and 58.46 ± 137.8 ng/mL in tocilizumab and control groups, respectively (*p =* 0.417 and *p =* 0.683).

**Figure 4:**
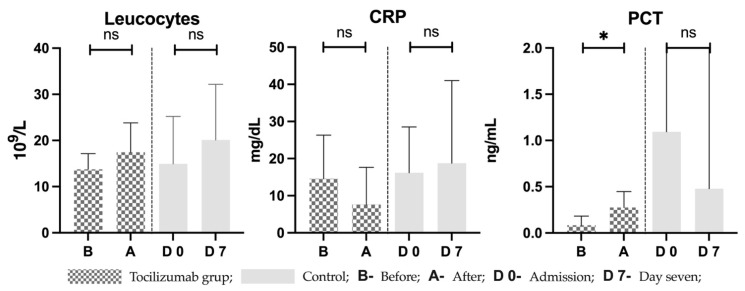
Laboratory parameters related to sepsis in the treated group and controls. B—right before receiving treatment with tocilizumab; A—seven days after treatment; D 0—on admission in control group; D 7—seven days after admission in controls; CRP = C reactive protein; PCT = procalcitonin; * = statistically significant; ns = statistically non-significant.

Other laboratory parameters such as leucocytes registered a positive trend in both groups with a mean difference of 3.73 ± 7.214 10^9^/L in the treated group and 5.17 ± 7.66 10^9^/L in controls (*p* = 0.26 and *p* = 0.52, respectively). CRP followed a decreasing trend after treatment, with a mean difference of −6.928 ± 11.46 mg/dL (*p* = 0.12), and an increasing trend in controls where a mean difference of 2.59 ± 11.19 mg/dL was observed. Finally, procalcitonin was statistically significant in the treated group, with a difference of 0.19 ± 0.12 ng/mL (*p* = 0.011), while in controls PCT decreased by a difference of −0.62 ng/mL. Before and after values in both groups are seen in Figure 4.

## 4. Discussion

### 4.1. COVID-19 May Produce Cytokine Storm and Persistent Inflammation

SARS-CoV2 activates T lymphocytes, specifically CD4, which become T-helper 1 cells (Th1). Th1, by means of granulocyte macrophage colonies stimulating factor (GM-CSF) and IL-6, go on to trigger CD14+ and CD16+ monocytes to become macrophages [15]. Matured monocytes further amplify and release cytokines like IL-6 to maintain a hyped inflammatory state and recruit more cells. Both types of cells have been found in higher titers in ICU patients compared to healthy subjects and are thought to directly target infected type II pneumocytes. The release of cytokines and direct targeting of infected cells produce an auto-maintained organ damage [16].

**Figure 5:**
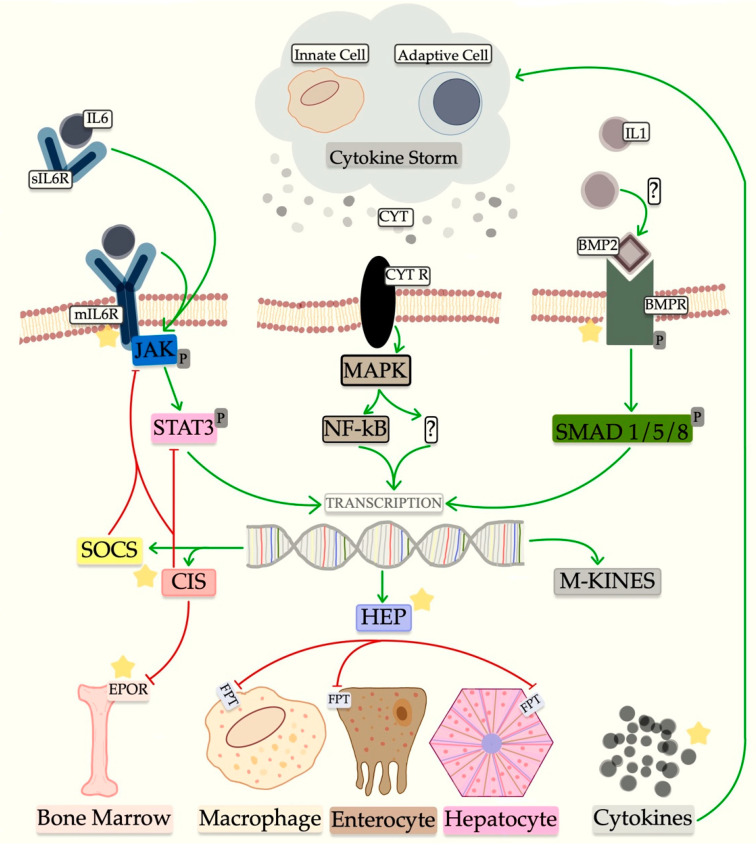
Cytokine storm. An array of cytokines is produced because of the viral insult. Most important cytokines are believed to be IL-6 and IL-1 with their respective signaling mechanisms. Alternatively, the MAPK/NF-κB pathway could play a role, but the cytokines involved are less known. In the end, anemia, hyperferritinemia and cytokine storm ensue. Receptors and their respective signaling mechanisms as well as their downstream products could represent potential therapeutic targets. Potentially, essential therapeutic targets are marked with yellow star and include IL-6, IL-1, BMP2 and BMPR, JAK, STAT3, SOCS, CIS, EPOR, CYT and CYT R, MAPK, NF-κB, SMAD1/5/8, HEP; IL-6 = interleukin 6; IL-6R = interleukin 6 receptor; JAK = Janus kinase; *p* = phosphorus; STAT3 = signal transducer and activator of transcription 3; SOCS = suppressor of cytokine signaling proteins; CIS = cytokine inducible SH2; EPOR = erythropoietin receptor; CYT = cytokine; CYT R = cytokine receptor; MAPK = mitogen activated protein kinase; NF-κB = Nuclear factor kappa B; ? = unknown mechanism; HEP = hepcitin; FPT = ferroportin; IL-1 = interleukin 1; BMP2 = bone morphogenic protein 2; BMPR = bone morphogenic protein receptor; SMAD1/5/8 = mothers against decapentaplegic family protein 1/5/8; M-KINES = monokynes.

Monocytes are continuously being activated while lymphocytes decrease in severe cases [17]. The latter could hinder the ability of cytotoxic cells to clear the virus. The neutrophil to lymphocyte ratio may possess prognostic values and is not limited to COVID-19 alone [18]. Persisting virulence and continuous cytokine release might be the cause for the cytokine storm seen in critically ill patients [10]. Figure 5 illustrates known and potential mechanisms involved in this immunological response. In cytokine storm, IL-6 and IL-1 are released among other cytokines such as tumor necrotizing factor alpha (TNF-α) and interferon gamma (IF-γ), to name a few [19]. It is believed that IL-6 plays the main role in maintaining inflammation via the Janus kinase/Signal transducer and activator of transcription 3 (JAK/STAT3) and mitogen activated protein kinase/nuclear factor kappa B (MAPK/NF-κB) pathways [20]. The complexity behind CS perhaps suggests why blocking the IL-6 route may not be enough to curb inflammation.

So far, meta-analysis and randomized control trials showed limited benefits of tocilizumab [21,22]. Despite many patients included in the analysis, the quality of evidence was, at best, moderate [23]. In our study, the small number of participants does not allow for a significant difference to be observed between groups, which limits the results to observing the trends. Given the rate of death in controls was 6.5 times the rate in the treated group, it is possible that tocilizumab may exert a positive effect on the outcome. However, it must be noted that from a clinical aspect our results indicate a marginal improvement.

The persistent inflammation seen in CS produces a state of hyperferritinemia and is associated with ROS induced cell death called ferroptosis [24]. As a result, iron leaks from the cytoplasm of damaged cells in the form of ferritin plus free iron and could possibly maintain inflammation [25]. In controls the inflammatory parameters remained elevated over time, which may have produced iron overload as indicated by the high ferritin and hepcidin with low circulating iron. In the treated group, serum iron as well as TS were restored 7 days after treatment with tocilizumab, but no significant change was observed in ferritin and hepcidin. The findings in the treatment group could be explained by a partial or incomplete anti-inflammatory effect of tocilizumab caused by an insufficient blockade of the IL-6 receptor [19] or by inflammation crossing over from the main IL-6-JAK/STAT3 pathway to an alternative pathway like IL-1 [26]. In hepatocytes, the beta subunit of IL-1 (IL-1β) produces hepcidin transcription via the bone morphogenic protein 2 (BMP2) and small mothers against decapentaplegic (SMAD) 1, 5 and 8. The way in which IL-1 activates the BMP/SMAD way is unknown [27]. Alternatively, the permanent state of inflammation and subsequent ferroptosis, where ferritin and iron are released concomitantly, could also explain our findings.

Finally, SARS-CoV2 has been reported to be a hepcidin mimetic [28,29] and was believed to target hemoglobin [7], in which case tocilizumab could have produced the normalization of plasma iron without an effect on ferritin, hepcidin and anemia. Given the limited number of patients and cytokines determined, we can only formulate hypothesis for future studies addressing pathophysiological mechanisms.

### 4.2. Anemia Is Multifactorial

At baseline, despite marked inflammation and iron sequestration, anemia was not present in either group. Judging by the results presented by Guz et al., it would be expected that by blocking the IL-6 pathway, an elevation in serum iron and TS as well as a decrease in hepcidin and ferritin would follow [30]. This could have a positive impact on the occurrence of anemia and the need for transfusions [31,32]. The rate of anemia in controls was twice that of the treatment group while the rate of transfusions was the same, yet neither reached statistical significance. Furthermore, both groups developed anemia after seven days. In our study we observed that ferritin and hepcidin remained elevated, unlike the results reported by Guz et al. [30]. A lack of an effect by tocilizumab could have been caused by the persistent inflammation, maintained by an alternative mechanism. As a result, anemia of inflammation could have ensued. It is also possible that the lack of a difference between groups is attributed to the small number of participants.

CIS3, a cytokine inducible SH2 (CIS) protein, is inhibitory, and could be incriminated for the occurrence of anemia. Under normal conditions, erythropoietin (EPO) binds to the receptor (EPOR). The binding stimulates a member of the tyrosine kinase family, specifically JAK2 to donate a phosphate to the signal transducer and activator of transcription 5 (STAT5). JAK2/STAT5 functioning is therefore essential for erythropoiesis [33]. CIS3 binds to the EPOR and JAK2 and impairs the normal functioning of this mechanism. CIS3 is related to a series of cloned CIS proteins such as the first generation CIS1 and second-generation JAB/SOCS1/SSI1 [34]. In COVID-19 pneumonia, the CIS3 production by IL-6 potentially exerts negative feedback on inflammation. It works by inhibiting the JAK2/STAT3 pathway [35], but it is not specific to it. Inhibition of the JAK2/STAT5 signaling also occurs and prevents transduction in erythropoiesis. Since CIS3 is stimulated by other proinflammatory as well as anti-inflammatory cytokines, selectively blocking IL-6 with tocilizumab seems to not be enough to prevent anemia. Furthermore, a weak erythropoiesis is associated with low erythroferrone and unchecked hepcidin [36].

Critically ill patients are exposed to frequent blood sampling and extracorporeal blood filtration processes, which produce anemia via loss of iron and hemoglobin at the same time [37]. This type of anemia is a combination of iron deficiency and inflammation and could explain why curbing inflammation alone does not produce a protective effect.

As for transfusions in our ICU, the indications and triggers for administering blood products vary and are left to the interpretation of the caring doctor [38]. The cross-over on the Kaplan Meier curve indicates an uneven hazard rate produced by different physicians which may be improve by more precise protocols.

### 4.3. Limitations of Tocilizumab and Potential Alternative Therapies

Tocilizumab is a monoclonal antibody which acts by blocking both plasma and cell membrane bound IL-6 receptors. The anti-inflammatory effect of tocilizumab is produced by preventing IL-6 from binding to the soluble receptor. However, blocking the membrane IL-6 receptor, which produces an innate anti-inflammatory effect, might explain why most of the studies evaluating the effects of tocilizumab did not demonstrate significant results [20].

Short- and long-term effects of tocilizumab were evaluated in patients with Castleman disease (CD), where chronic elevation of IL-6 produces hepcidin-mediated inflammatory anemia. Positive short- and long-term effects were observed in terms of hepcidin downregulation while in terms of reversal of anemia, positive results were observed at the three-month time-point. IL-6 levels increased as tocilizumab was initiated due to the competitive nature of this antagonist [39]. In our study, IL-6 did not increase significantly at 7 days, possibly indicating an incomplete effect of tocilizumab, in which case a multiple dose regime might be warranted [40]. In terms of side effects profile, bacterial infections are the most relevant given the strong immunosuppression. In our study the median sepsis free time was not significantly different between groups but must be interpreted with the limitations of this study in mind.

Inhibition of signaling mechanism of IL-6 with JAK/STAT inhibitors proved efficient in autoimmune disease. In COVID-19 JAK inhibitors produce clinical and respiratory improvements; however, the antiviral cytokine IFN-α is also inhibited, which represents a downside, much like with tocilizumab [15].

Alternative therapies which target cytokines specifically are directed against IL-1, IL17, and TNF-α. Anakinra exerts its anti-inflammatory effects by binding to the IL-1R, which inhibits the interaction of both IL-1α and IL-1β with the receptor. IL-17 can be targeted directly with either IL-17A inhibitor secukinumab or IL-17R blocker brodalumab [41]. An observed high serum level of TNF-α in COVID-19 patients has indicated a potential role of targeting TNF-α [10,42]. Less selective therapies employed in COVID-19 have been chloroquine and hydroxychloroquine. These drugs have been used because of a theoretical ability to curb inflammation by targeting IL-6 and TNF-α. Today, this therapy is no longer recommended as they demonstrated limited clinical benefits [15]. Non-selective anti-inflammatory therapies such as corticosteroids have been very useful in patients with severe respiratory insufficiency requiring oxygen therapy with abundant clinical research to back it up [43]. Potential benefits could be observed with blood purification therapies as well, which eliminate, to an extent, many of the cytokines involved in CS [44,45]. This is employed in critical care, mainly as a last resort when coexisting indications for such procedures exist.

Nanoparticles (NPs) are versatile and have the potential to be adopted as alternative therapy [46]. Cuprous-ferric delafossite nanoparticles are molecules which have antibacterial as well as anti-inflammatory properties and could prove useful in the management of COVID-19 patients experiencing CS [47]. Critically ill patients are at risk of developing concomitant bacterial infections. Metal ion nanoparticles such as iron oxide and zinc oxide nanoparticles have good antibacterial properties and could be used as an adjuvant in the prevention or treatment of bacterial superinfections [43]. Furthermore, the ability of NPs to carry drugs could be adopted to neutralize the SARS-CoV2 [48]. Finally, NPs could be used for their gene carrying and targeting capabilities. The former allows for more efficient vaccines while the latter can be implemented for targeting of hepcidin by gene silencing.

So far, the studies which investigated these methods are limited to cell and murine experiments [49]. Recent reports present the synthesis of specific targeted hyaluronate recoated *N*,*N*,*N*-trimethyl chitosan nanoparticles aiming to deliver small interference RNAs for the blocking of the IL-6–STAT 3 mechanism. These promising results have only been achieved in cancer cell progression experiments; however, the mechanism is common with that of the COVID-19 cytokine storm and creates the premises for multiple applications [50]. The scientific opportunity was also validated by another group of researchers, which proposed a hyaluronate-gold nanoparticle/tocilizumab structure and reported significant effects in rheumatoid arthritis [51]. Application of this NP assembly in COVID-19 creates hope for an efficient approach to reduce inflammation. Usage of a specific peptide capable to inhibit the interaction between IL-6 and IL-6R as a functionalization element for nanoparticles has also been reported in combination with a specific oligoment DNA. The authors have demonstrated an antitumor effect achieved in glioma; however, the similarity in IL-6R coupling mechanisms has the potential to be extended to COVID-19 induced inflammation [52].

Another nano mediated application of described mechanisms was recently proposed. By fusing nanovesicles derived from genetically engineered cell membranes and human monocytes, the authors have produced an abundance of angiotensin-converting enzyme (ACE) and cytokine receptors on the engineered cells. Results show efficient absorption of viruses, IL-6 and GM-CSF inflammatory cytokines, with therapeutic effects in COVID-19 infections [53].

Innovative approach of synthetizing nanoparticles capable of slow release and generation of nitric oxide (NO) has also been reported, with significant results in decreasing IL-1α, IL-6 and TNF-α from monocytes [54]. Moreover, it has been demonstrated that green synthesis of gold nanoparticles using Euphrasia oficinalis inhibit TNF-α and IL-1ß secretion [55]. The recent wide interest in research on targeting IL-6 centered mechanisms may generate future studies on novel alternatives to current therapeutic options. It could produce an increase in the effects of drugs such as Tocilizumab or even outperform them.

### 4.4. Study Limitations

The small number of patients included in this study limits the ability to draw significant conclusions. As a pilot study it was solely meant to use data collected from several subjects in each group as a calculus basis for prospective sample size estimation. Based on presented IL-6 data, for a level of 80% for study power and a level of significance of 5%, the present study would require a sample size of 160 subjects for each study group for appropriate validation of results. Furthermore, by looking at trends of results it is possible to gain ideas for future studies and therapeutic approaches for excessive inflammation.

For hepcidin and IL-6, we used ELISE kits intended for research only and there are no reference values provided by the manufacturer. As a result, we cannot use the values in our paper in clinical practice and make clinical decisions based on them. In our paper we used the reference values from similar studies.

## 5. Conclusions

Anemia is multifactorial, produced by humoral and external factors concomitantly. Addressing this issue requires further understanding of the alternative pathways of hepcidin induction. Our results showed that new therapeutic approaches are needed since blocking what was supposed to be the main pathway of inflammation did not have an effect downstream. This might be explained by the multitude of interleukins present in cytokine storm, requiring new and innovative approaches. This study is unique because, to our knowledge, this is the only study so far which looked at the results of blocking the main inflammation pathway on anemia and transfusions.

## Figures and Tables

**Table 1:** Baseline characteristics overall (general), patients with Tocilizumab (treatment) and their matched controls respectively (controls).

	General	Control (C)	Treatment (T)	Normal Value	*p*-Value (C and T)
	Patients				
Total	12	6	6	-	-
Female n (%)	8 (67)	4 (67)	4 (67)	-	0.99
Male n (%)	4 (33)	2 (33)	2 (33)	-	
	Mean (±SD) or Median (range)				
Age (years)	67 (±13)	67.83 (±11.82)	66.17 (±14.77)	-	0.8335
Charlson	3 (±1)	3.500 (±1.38)	3.333 (±1.97)	-	0.8684
APACHE II score	13 (±8)	11.33 (±5.72)	15.50 (±10.23)	-	0.4043
Hemoglobin (g/dL)	13.37 (±0.6)	13.47 (±0.62)	13.27 (±0.62)	Female: 12–15.5	0.5868
				Male: 13–17	
MCV (fL)	87.85 (±2.5)	87.35 (±2.29)	88.35 (±2.8)	80–95	0.5139
TS (%)	17.67 (±10.63)	17.00 (±9.84)	18.33 (±12.27)	16–45	0.8397
Transferrin (mg/dL)	139.6 (±48.22)	154.3 (±65.77)	125.0 (±16.49)	200–360	0.3143
Ferritin (ng/mL)	969.5 (650.8–2199)	1145 (595–2587)	913 (675.8–1695)	15–200	0.9372
Iron (µg/dL)	31.67 (±18.65)	29.83 (±12.94)	33.50 (±24.28)	65–180	0.7508
Hepcidin (pg/mL)	247.7 (±69.72)	258.7 (±103.5)	238.6 (±30.55)	*	0.6580
IL-6 (pg/mL)	147.5 (36.15–372.8)	83.40 (15.10–884.3)	178.3 (103.5–295.6)	*	0.4500
Leucocytes (10^9^/L)	13.56 (±7.19)	14.94 (±10.29)	12.18 (±1.83)	<14	0.5332
Neutrophiles (10^9^/L)	12.13 (±7.61)	13.52 (±10.87)	10.74 (±2.15)	1.5–7.4	0.5532
Lymphocytes (10^9^/L)	0.87 (0.37–1.23)	0.5850 (0.3–1.46)	1.130 (0.66–14.19)	1.5–3.5	0.3095
Thrombocytes (10^9^/L)	303.9 (±92.41)	254.7 (±54.69)	353.2 (±99.87)	150–380	0.0601
CRP (mg/dL)	15.17 (±11.60)	16.18 (±12.35)	14.15 (±11.88)	<0.5	0.7780
PCT (ng/mL)	0.15 (0.05–1.58)	1.095 (0.11–12.73)	0.11 (0.05–0.18)	<0.5	0.2597
ALAT (U/L)	34 (±8.79)	30.50 (±3.017)	37.50 (±11.47)	<35	0.1788
Albumin (g/dL)	2.82 (±0.55)	2.8 (±0.79)	2.84 (±0.19)	3.5–5	0.9067
Fibrinogen (mg/dL)	524.6 (±124.0)	494.3 (±147.3)	555.0 (±99.44)	200–400	0.4223
Bilirubin (mg/dL)	0.55 (±0.18)	0.58 (±0.21)	0.53 (±0.16)	<1.2	0.6683
Creatinine (mg/dL)	1.0 (±0.561)	1.21 (±0.7041)	0.79 (±0.3022)	<1.2	0.2090
LDH (U/L)	483.6 (±188.8)	385.0 (±57.82)	582.2 (±227.4)	<250	0.0666
Lipase (U/L)	32.42 (±15.60)	34.0 (±18.34)	30.83 (±13.89)	<67	0.7430

SD = Standard deviation; APACHE II score = Acute Physiology and Chronic Health Evaluation II score; MCV = Mean corpuscular volume; TS = Transferrin saturation; IL-6 = Interleukin 6; CRP = C reactive protein; PCT = Procalcitonin; ALAT = Alanine transaminase; LDH = Lactate dehydrogenase; * = values vary depending on studies.

## Data Availability

Not applicable.

## Appendix A. Criteria for Defining Severe and Critical Illness

Severe Cases
pulse oximetry reading < 94% (with supplemental oxygen) OR arterial oxygen pressure to fraction of inspired oxygen ratio (P/F) below 300
elevated respiratory rate (>30 breaths per minute) and dyspnea
>50% lung infiltrate
Critically ill
respiratory insuficiency
cardio-circulatory instability requiring pressor support OR multiple organ failure
